# Three taphonomic stories of three new fossil species of Darwin wasps (Hymenoptera, Ichneumonidae)

**DOI:** 10.1038/s41598-024-67466-z

**Published:** 2024-07-29

**Authors:** Alexandra Viertler, Fons Verheyde, Martin Schwarz, Georg Schulz, Seraina Klopfstein, Bastien Mennecart

**Affiliations:** 1 Institute of Ecology and Evolution, University of Bern, Baltzerstrasse 6, 3012 Bern, Switzerland; 2 Natural History Museum Basel, Augustinergasse 2, 4051 Basel, Switzerland; 3 Aartshertoginnestraat 58/01, 8400 Ostend, Belgium; 4 Biologiezentrum Linz, OÖ Landes-Kultur GmbH, Johann-Wilhelm-Kleinstrasse 73, 4040 Linz, Austria; 5 Department of Biomedical Engineering, Core Facility Micro- and Nanotomography, University of Basel, Hegenheimermattweg 167 B/C, 4123 Allschwil, Switzerland; 6 Department of Biomedical Engineering, Biomaterials Science Center, University of Basel, Hegenheimermattweg 167 B/C, 4123 Allschwil, Switzerland

**Keywords:** Palaeontology, Taxonomy, Zoology

## Abstract

Amber captures a snapshot of life and death from millions of years in the past. Here, the fate of three fossil Darwin wasps in Baltic amber is virtually dissected with the help of micro-CT scanning, to better understand the taphonomic processes that affected their preservation. The states of the fossils range from nearly perfect preservation, including remains of internal organs, to empty casts that were strongly affected by decomposition. We describe the three specimens as new taxa, *Osparvis aurorae* gen. et sp. nov., *Grana harveydenti* gen. et sp. nov. and *Xorides*? *romeo* sp. nov. Based on the taphonomic and morphological interpretations, we conclude that two specimens were trapped alive, and the third ended up in resin post-mortem. The morphology and classification of the specimens provide clues regarding their ecology, and we discuss their likely hosts and parasitation modes. Taken together, our three wasp fossils showcase how an integrative analysis of amber taphonomy, taxonomic association and morphology can shed light onto past biodiversity and offer valuable insights for interpreting their evolutionary history.

## Introduction

Amber inclusions from different geological epochs serve as windows to the past, revealing a long-gone and fascinating diversity of various organisms, especially insects. An extraordinary feature of amber is that it captures a momentary picture of life and death. Traces of movement within the amber indicate that the specimen was still alive when it first touched the resin^1^. When an insect gets covered by resin, its preservation and fossilisation over time are influenced by various physiochemical and biological processes. The study of these fossilization processes is the topic of taphonomy. Experiments have been conducted to understand the preservation of insects in amber, highlighting the impact of factors such as time, dehydration, gut microbiota, and the embedding substrate^2,3^.

But which insects get stuck in resin? The viscosity of the resin and the season in which it is produced, along with the behaviour of the insects, influence which organisms get entrapped^1,4^. Smaller insects are likely to be covered entirely and then succumb to asphyxiation, while larger insects rarely get trapped entirely by a single resin flow. They may instead either manage to free themselves and escape or die due to predation or exhaustion^1^. Apart from size, certain insects face a higher risk of ending up in resin than others, like xylophagous species or those that inhabit tree bark^1,5,6^. Parasitoid wasps (Hymenoptera) that attack insects in the proximity of wood (e.g. parasitoids of wood-boring hosts) are particularly prone to getting stuck inresin^6,7^.

The morphology of a parasitoid wasp often offers valuable insights into its ecology. For instance, large and robust mandibles indicate an adaptation of a species to freeing itself from the substrate in which it was encased during the pupal stage^8^, while a stout body and short antennae in female wasps can be an adaptation for searching for a host in decaying wood or under the ground^9^. In fossils, we very rarely encounter direct evidence of a connection between a parasitoid and its host, such as when a parasitoid or its larva is embedded inside or with its host^10–13^. More frequently, we try to infer a parasitoid’s host group with the help of its morphology. Accordingly, the diversity in morphological adaptations found in parasitoids can reflect a diverse host range.

One group of parasitoid wasps that exhibits an astonishing diversity of both hosts and morphology is the Darwin wasps (Ichneumonidae). They belong to the Hymenoptera, the third most abundant arthropod order in Baltic amber^14^. Darwin wasps are especially frequently found in Baltic amber, which is the largest amber deposit on Earth^15^, but the vast majority of species remain undescribed. Manukyan and Zhindarev^16^ gave an overview of the species found in Baltic amber and concluded that over 90% of Darwin wasps belong to only four subfamilies, Phygadeuontinae, Hybrizontinae, Townesitinae, and Pherhombinae, the last two being extinct today. In Phygadeuontinae, the predominant ichneumonid subfamily in Baltic amber, only one species has been described so far^17^. In total, only ten of the 45 subfamilies recognized today have been reported from Baltic amber, alluding to the strong taxonomic bias imposed by amber taphonomy.

In this study, we describe two new fossil genera and species of Phygadeuontinae, and one new fossil Xoridinae, a subfamily here reported from amber for the first time. Taphonomic processes are evaluated to uncover the circumstances of the deaths of the three wasps in amber. Through detailed examination, we gather information from the amber piece itself, its eusyninclusions^18^, the wasps’ body position, signs of destruction or decay, and internal structures revealed by micro-CT scans to elucidate the probable cause of death of each fossil individual. We then carefully integrate our findings with paleoecological inferences based on the wasps’ morphology and relationships to extant taxa, in order to provide a more complete picture of the parasitoid wasp diversity in the resinous forest, that produced Baltic amber.

## Results

### Systematic palaeontology

We here provide, for each fossil, an outline of its systematic placement and brief diagnoses of the new genera and species. In-depth discussions of the classifications and detailed descriptions of the new taxa are provided as supplementary material (Supplementary File S1).

**Hymenoptera** Linnaeus, 1758.

**Ichneumonidae** Latreille, 1802.

Subfamily **Phygadeuontinae?** Förster, 1869.

Genus ***Osparvis*** Viertler, Schwarz, Verheyde et Klopfstein, gen. nov.

**ZooBank**: urn:lsid:zoobank.org:act:6527F151-3165-40CC-A3C4-D69367981A38.

**Type species:** *Osparvis aurorae* gen. et sp. nov.

**Etymology:** Combines the words “os” and “parvum” in Latin, meaning “small mouth” due to the very small mandibles and small clypeus of the specimen. Gender: masculine.

*Systematic placement* This fossil is difficult to place in a Darwin wasp subfamily, as it combines several plesiomorphic features with characters that are very rare or even unique in Darwin wasps. The ancestral characteristics, include the outward-bowed vein 2 m-cu with two bullae in the forewing, the completely areolated propodeum, and finally the medium-length ovipositor with nodus and ventral ridges. These traits were already present in wasps from the Cretaceous^19^. The small clypeus, exposed labrum and long malar space are rare characters, present in only a few species (compare^20^). The rather broadly attaching first tergite with the spiracle slightly posterior to the middle and a large laterotergite, on the other hand, are unique in Darwin wasps. While the fossil does not fit in any of the known subfamilies completely, at least there is no strong evidence against Phygadeuontinae, where we place it with some uncertainty.

*Genus diagnosis* The new genus is unique in having a large and sclerotized first laterotergite and an exposed labrum, a combination not found in any extant phygadeuontine species. It combines the following characters: Thickened antenna, strongly tapered and short mandibles, bilobed clypeal apex, first tergite short and anteriorly wide, areolet open.

**Figure 1:**
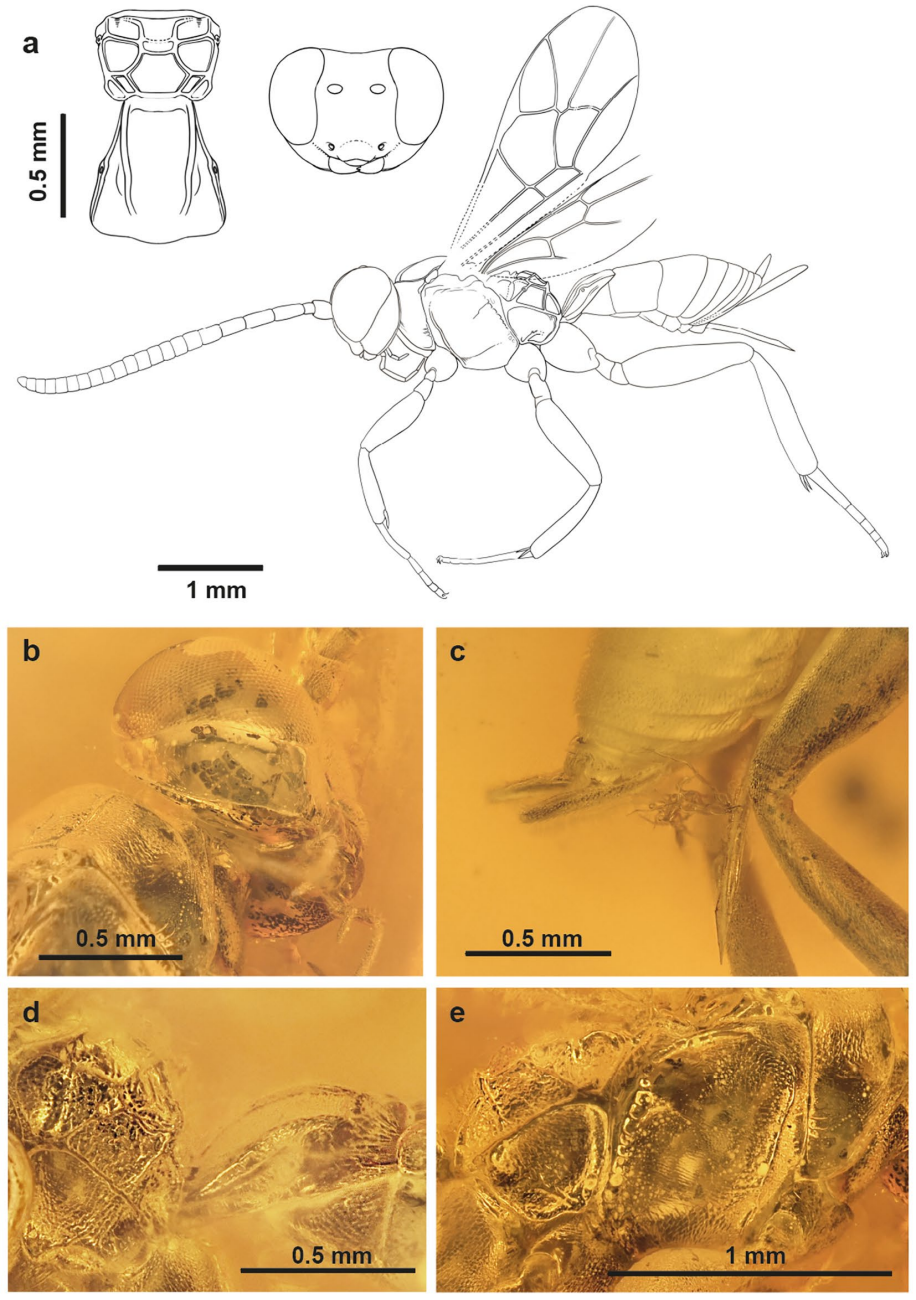
Holotype of *Osparvis aurorae* gen. et sp. nov. (NMB F3847). (**a**) Interpretative drawing with additional drawing of the propodeum and first tergite in dorsal view, and head from front view. (**b**) Photo of head, ventrolateral view. (**c**) Photo of apical part of metasoma with ovipositor, lateral view. (**d**) Photo of propodeum and first tergite, lateral view. (**e**) Photo of thorax, lateral view.

*Osparvis aurorae* Viertler, Schwarz, Verheyde et Klopfstein sp. nov. (Fig. 1).

**ZooBank**: urn:lsid:zoobank.org:act:CCB60345-F4D1-4B24-B06C-75CDCA65A9AA.

**Etymology**: Named after the fictional character Aurora from the Walt Disney movie “Sleeping Beauty”, since this specimen is beautifully preserved.

**Type specimen**: Holotype: female (NMB F3847). Baltic amber. Provenance: Kaliningrad region (Yantarny, Russia), Late Eocene. Deposited in Switzerland, Natural History Museum Basel. Eusyninclusion of some oak flower stellate hairs (trichomes).

**Type condition**: Very well preserved, including some internal structures and muscles.

**Diagnosis**: See genus diagnosis.

Description of *Osparvis aurorae* gen. et sp. nov. is available in Supplementary file S1.

Subfamily **Phygadeuontinae** Förster, 1869.

Genus ***Grana*** Viertler, Schwarz, Verheyde et Klopfstein, gen. nov.

**ZooBank**: urn:lsid:zoobank.org:act:A1D71E27-639C-4C4F-AFB1-64207D0E3244.

**Type species:** *Grana harveydenti* gen. et sp. nov.

*Etymology* Short form of the Latin adjective “granulosus”, which emphasizes the overall granulate body sculpture of the specimen. Gender: feminine.

*Systematic placement* The combination of the fossil’s body characteristics clearly points to Phygadeuontinae, with the presence of a sternaulus that ends above the posterior ventral edge of the mesopleuron, the fore wing with a pentagonal areolet and two bullae on vein 2 m-cu, the complete carination of the propodeum, the petiolate first tergite with the spiracle behind the middle, and the rather long ovipositor^20^.

*Genus diagnosis* This genus is distinct from other genera in Phyadeuontinae in having a slight oblique truncation of the scape, no visible tooth or tubercles on the apical margin of the clypeus, a normal length of the maxillary palps, a closed areolet, a relatively short 2R1 cell, an intercepted nervellus, a distinct area superomedia that is around as long as wide, and a sclerotized part of the first sternite ending slightly before the spiracle.

**Figure 2:**
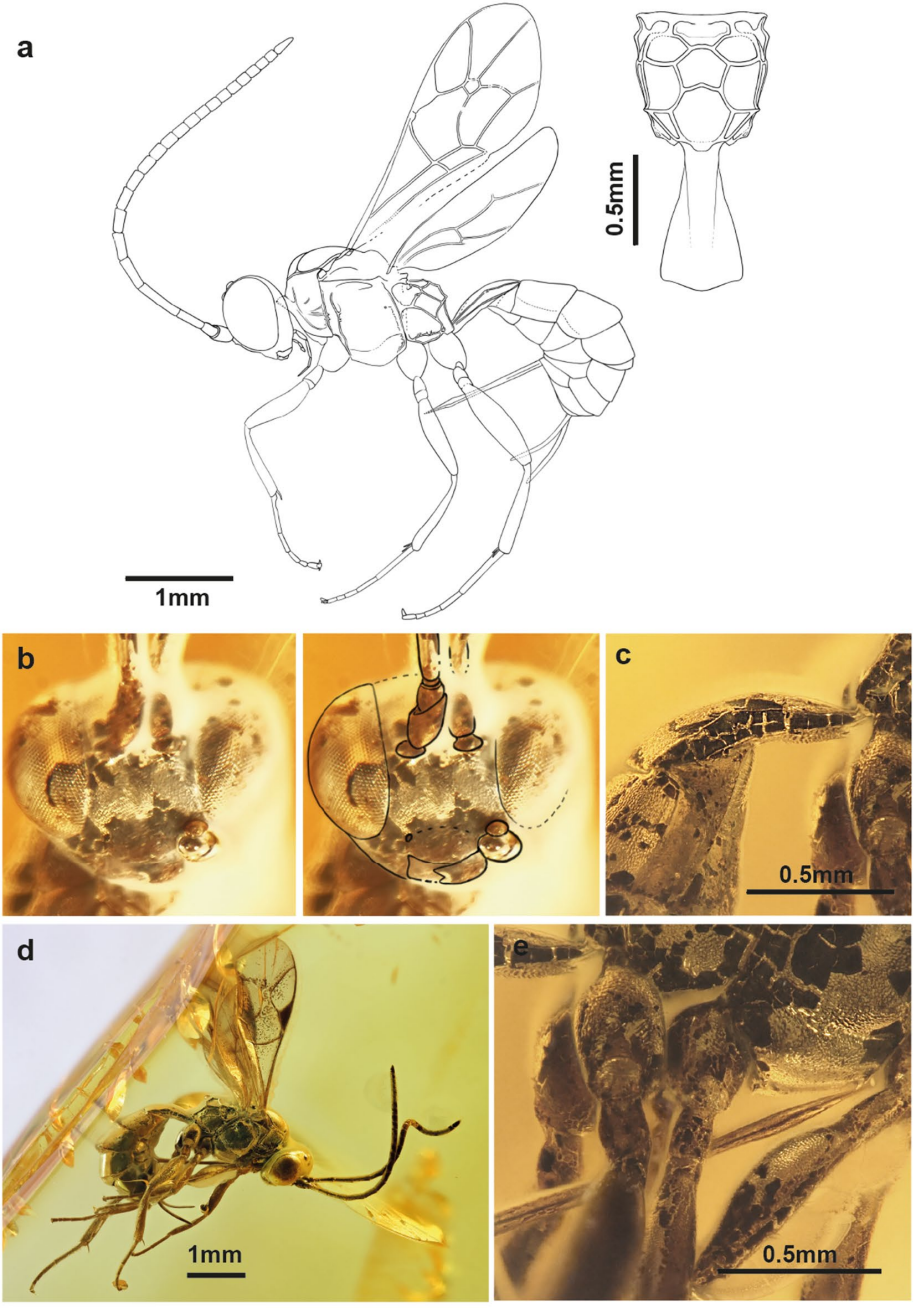
Holotype of *Grana harveydenti* gen. et sp. nov. (NMB F3848). (**a**) Interpretative drawing with additional drawing of the propodeum and first tergite in dorsal view. (**b**) Photo of face, in front view. On the right side the outlines of facial structures are shown, including an air bubble. (**c**) Photo of first tergite, lateral view. (**d**) Photo of holotype, lateral view. Photo by Jonas Damzen. (**e**) Photo of ovipositor and ventral side of thorax, lateral view.

*Grana harveydenti* Viertler, Schwarz, Verheyde et Klopfstein, sp. nov. (Fig. 2).

**ZooBank**: urn:lsid:zoobank.org:act:D9592537-3ED5-4D69-A981-C0C074BB2CE0.

**Etymology**: Named after the fictional supervillain Harvey Dent (also called Two-Face), from the DC-Comic Batman. Like this character, this specimen has one side of its body destroyed, while the other side is intact.

**Type specimen**: Holotype: female (NMB F3848). Baltic amber. Provenance: Kaliningrad region (Yantarny, Russia), Late Eocene. Deposited in Switzerland, Natural History Museum Basel. Eusyninclusions of a mite and various plant matter in debris.

*Type condition*: Right side well-preserved, left side opaque and covered in bubbles and white coatings. Right fore wing is more or less flat, left fore wing and hind wings are slightly folded. Metasoma is bent inwards, ovipositor almost touching mesopleuron.

**Diagnosis**: See genus diagnosis.

Description of *Grana harveydenti* gen. et sp. nov. is available in Supplementary file S1.

Subfamily **Xoridinae** Shuckard, 1840.

**Genus** *Xorides*? Latreille, 1809.

**Figure 3:**
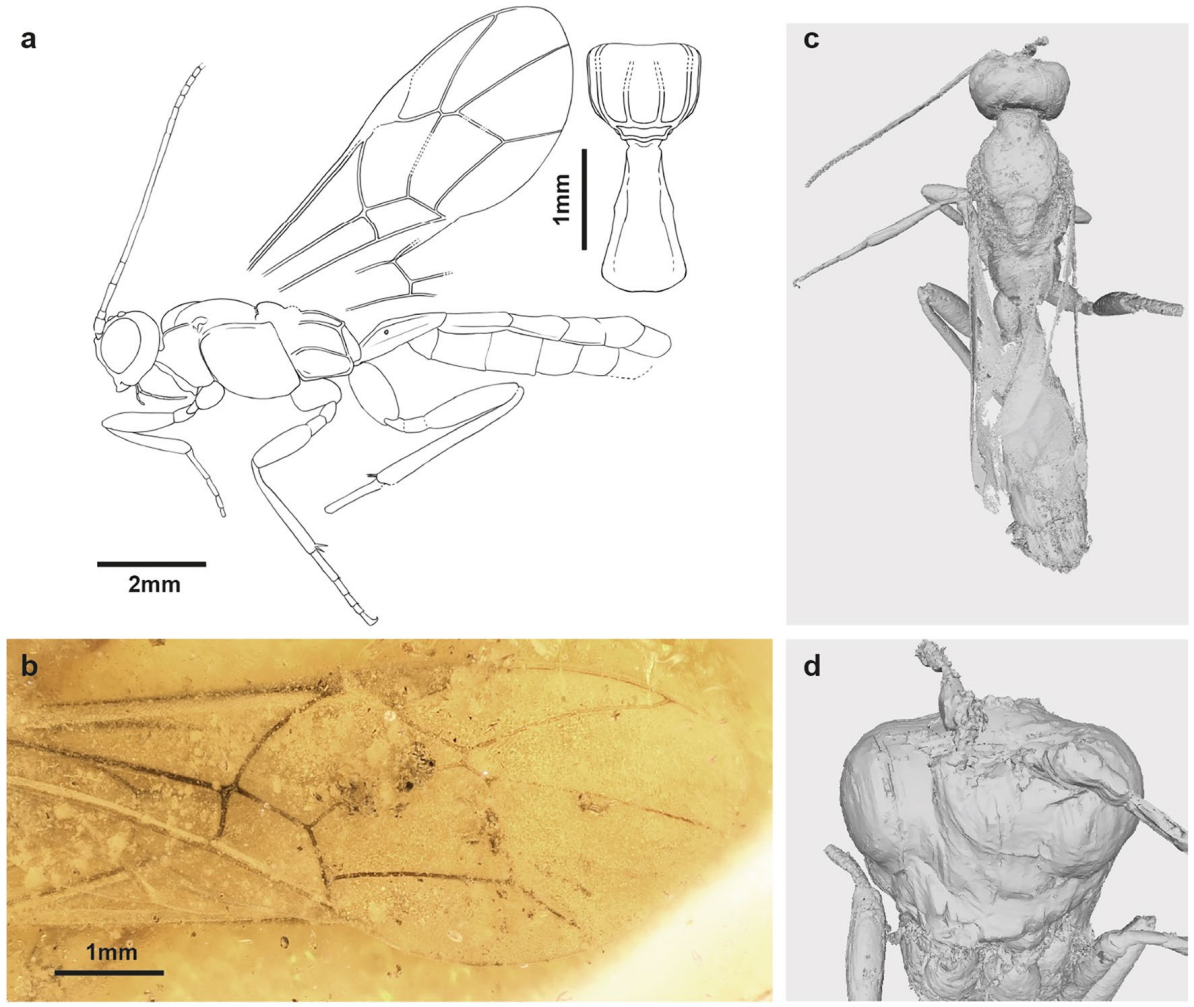
Holotype of *Xorides*? *romeo* sp. nov. (NMB F3849) (**a**) Interpretative drawing with additional drawing of the propodeum and first tergite in dorsal view. (**b**) Photo of fore wing. (**c**) Whole body in dorsal view, after micro-CT scanning. (**d**) Reconstructed face in front view, most parts indiscernible.

***Xorides? romeo*** Viertler et Klopfstein, sp. nov. (Fig. 3).

**ZooBank**: urn:lsid:zoobank.org:act:1243A9AC-CAC2-4823-A142-175177EB0C37.

**Etymology:**: Named after the male protagonist Romeo Montague from Shakespeare's Romeo & Juliette.

**Type specimen**: Holotype: male? (NMB F3849). Baltic amber. Provenance: Kaliningrad region (Yantarny, Russia), Late Eocene. Deposited in Switzerland, Natural History Museum Basel.

**Type condition**: Poorly preserved. Specimen covered in various debris and bubbles, hiding most of the body dorsally and ventrally. Antennae incomplete; face covered with dense miniscule bubbles and largely disintegrated. Dorsal view on mesopleuron covered by dense, fine bubbles and opaque foam. Apex of all legs missing except for one mid leg, which has the claw well visible. Metasoma dorsally covered by very well-preserved fore and hind wings. Apical part of metasoma missing.

*Systematic placement* The elongate body shape, together with the wide gena, and relatively small eye point to a wood-boring subfamily. The antefurcal 1cu-a on the fore wing is not common but occurs in some subfamilies of the informal group pimpliformes (i.e., Poemeniinae, Acaenitinae, Diacritinae, Rhyssinae, Pimplinae) and in the subfamily Xoridinae. However, many of those subfamilies can be excluded by specific characteristics that are different or absent in the fossil (e.g. transverse rugae on mesoscutum in Rhyssinae), or their rather stout appearance (Pimplinae). The fossil has simple claws, rather extensive propodeal carination, an open areolet, a straight 2 m-cu, and its nervellus intercepted below the middle, which excludes Poemeniinae, Acaenitinae, and Diacritinae. The elongate body, slightly swollen femur, very short 2Rs vein, and rather elongate first tergite point to Xoridinae.

*Diagnosis* Some important characters are not visible due to the preservation of the fossil, which would confirm the placement in the extant genus *Xorides*. We can neither see chisel-shaped mandible nor oblique grooves on the laterotergite of the second tergite, as would be the case in *Xorides*. Therefore, we place this fossil species only tentatively in the genus *Xorides*. This species is unique in fossil *Xorides* with the combination of slender hind femur and tibia, and wing venation characteristics like the 1cu-a antefurcal to 1 M&Rs, 2cu-a shorter than 3Cu, and a sinusoidal 4Rs. The description of *Xorides*? *romeo* sp. nov. is available in Supplementary file 1.

### Specimens’ preservation

**Figure 4:**
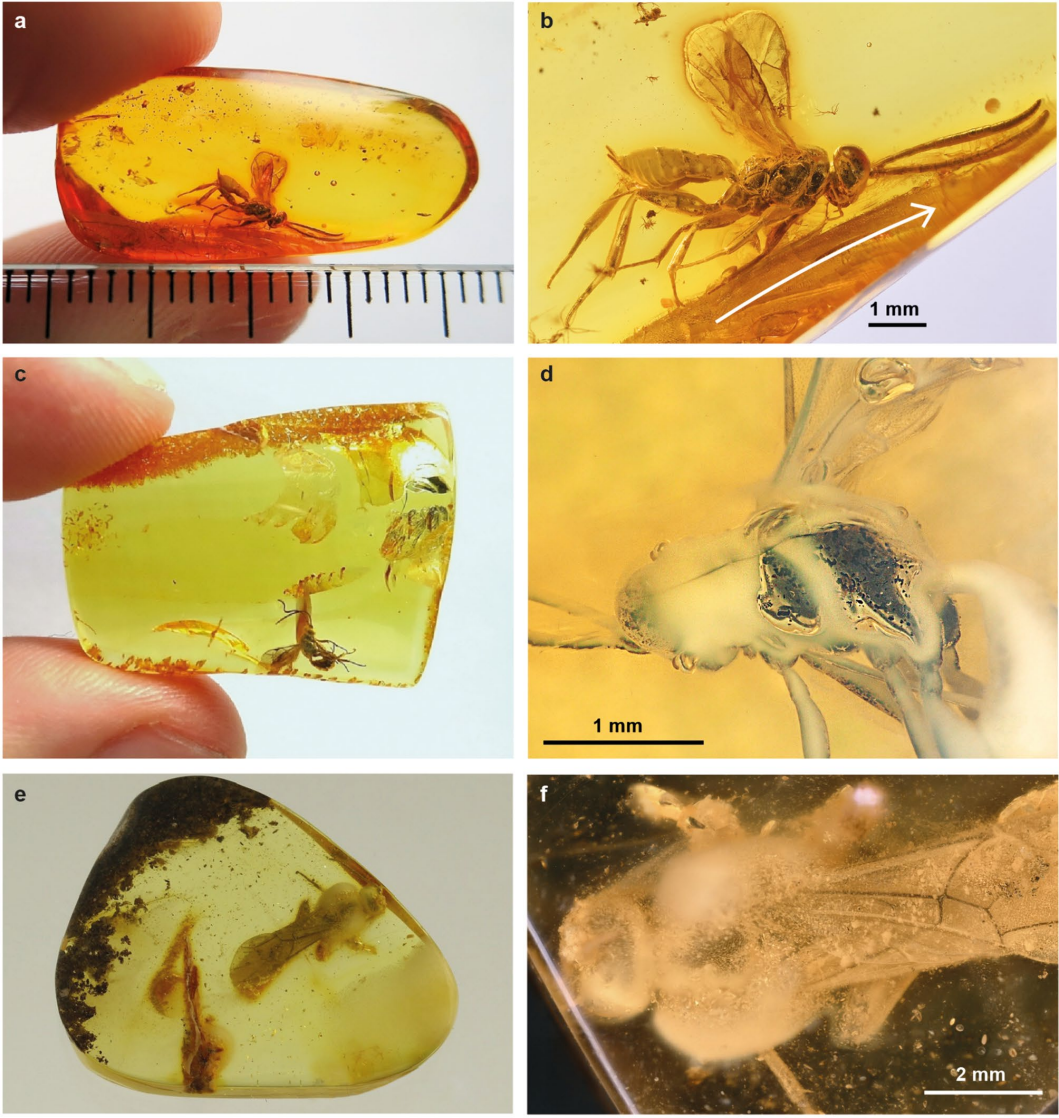
The three amber pieces and their ichneumonid inclusions, showcasing different qualities of preservation. (**a**–**b**) well-preserved *Osparvis aurorae*, white arrow indicates a second resin flow; (**c**–**d**) partly decomposed *Grana harveydenti,* with extensive milky coatings and large bubbles on one lateral side*;* (**e**–**f**) *Xorides*? *romeo,* with various bubbles, milky coatings, and debris around the body. Photos (**a**–**c**) by Jonas Damzen, and (**e**) by Marius Veta.

**Figure 5:**
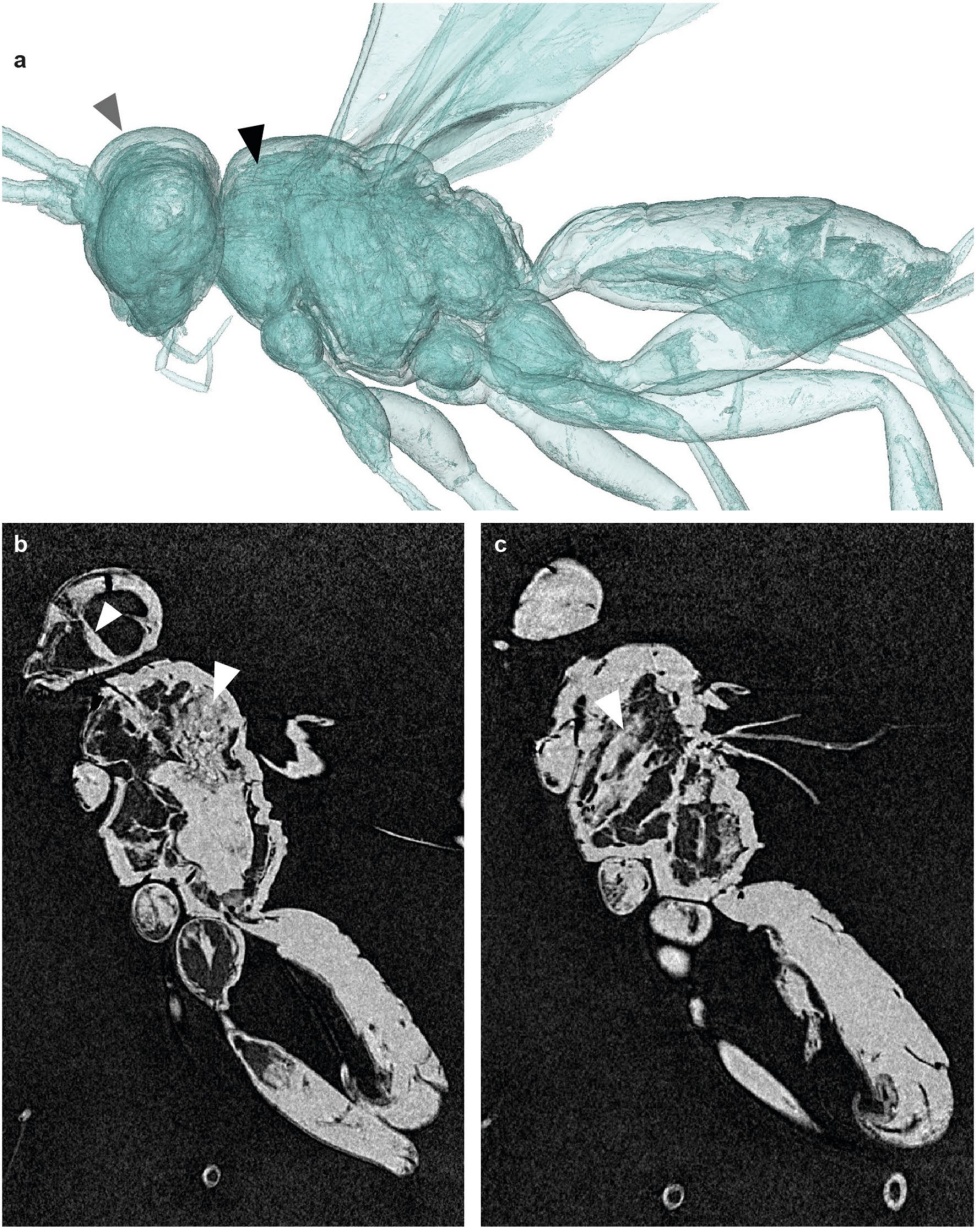
Taphonomy of *Osparvis aurorae* gen. et sp. nov. (**a**) Outer cuticle (transparent blue, grey triangle) and inner structures (opaque blue, black triangle). (**b**, **c**) Example lateral slides of the micro-CT scan with preserved muscle tissues (white triangles); in (**b**) with a structure reaching to antennal attachment on the head; in (**c**) muscles spanning from ventral to dorsal side of thorax.

The amber piece of *Osparvis aurorae* (Fig. 4a–b) measured 22 × 10 × 5 mm before cutting it for the scanning procedure, is bright orange in colour and contains many oak trichomes. The wasp was covered in one flow or drop, but we can clearly see a second resin flow. The direction of the resin flow within the amber piece surrounding *O*. *aurorae* is not readily apparent. However, upon closer examination, a subtle movement of resin is discernible in front of the specimens’ head. There is clear evidence of movement in the amber around the legs and wing base indicated by presence of round smears. The specimen’s body and antennae are in a natural position, where the antennae are forward, and legs backward oriented (Fig. 4a,b). On one side of the wasp there are some small bubbles present and the spiracles on the tergites show some milky coatings. However, the wasp is unharmed. The micro-CT scan revealed that this specimen has mostly intact internal structures, including muscle tissues (Fig. 5, Supplementary Video S2).

**Figure 6:**
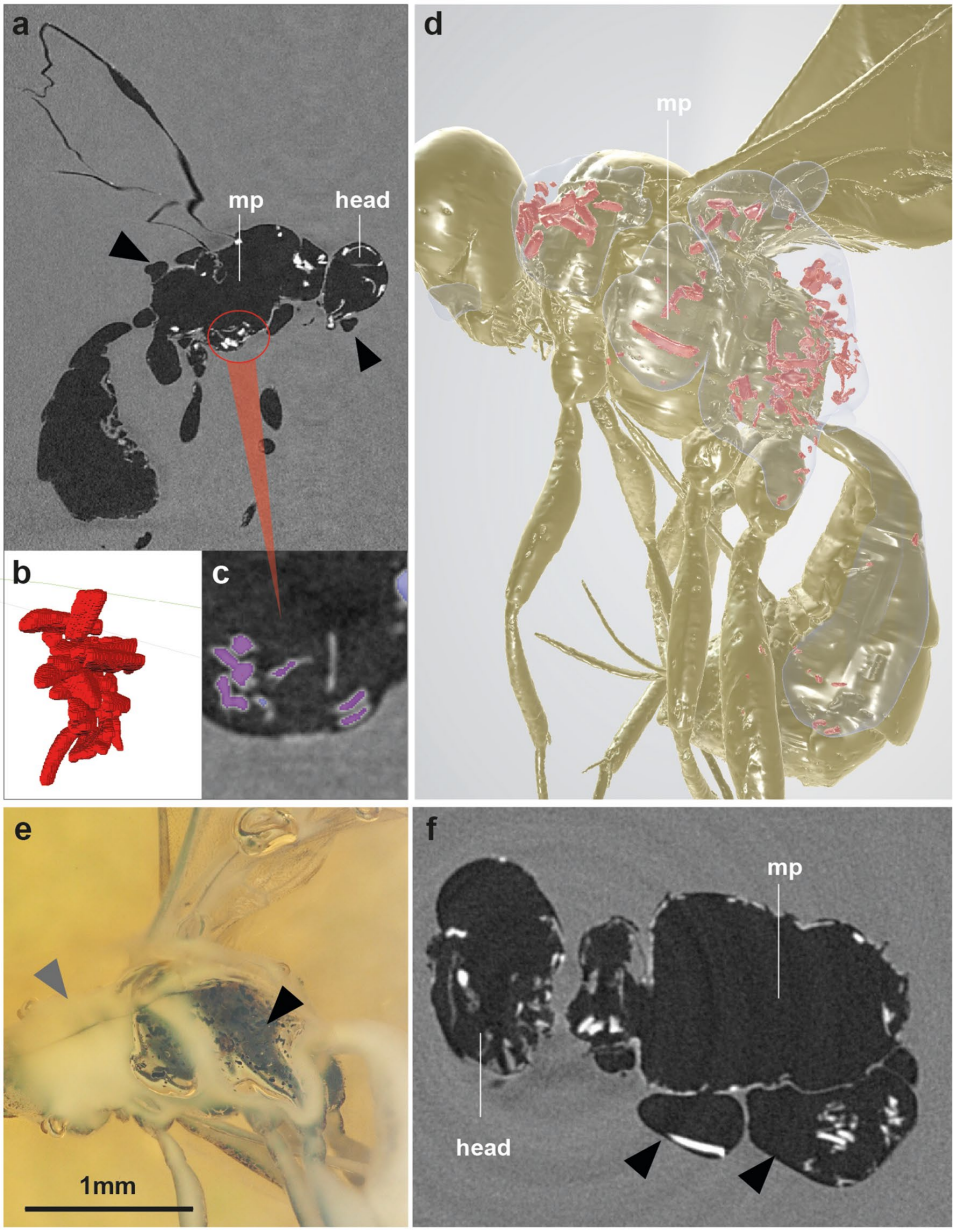
Taphonomy of *Grana harveydenti* gen. et sp. nov. (**a**) Example slide of micro-CT scan of the specimen in lateral view. (**b**–**c**) Elongated structures found along this ventro-lateral side. (**d**) Reconstructed wasp, showing the lateral side with bubbles and rod-like structures (red). (**e**) Photo of the opaque lateral side with milky coating (grey triangle) and bubbles with dark spots (black triangle). (**f**) Slide of micro-CT of the specimen in dorsal view showing the head and the mesopleuron, with the well-preserved right side of the wasp above and the more decomposed, left side below. The rod-like structures are positioned within the bubbles (black triangles). Mesopleuron (mp).

The amber piece of *Grana harveydenti* (Fig. 4c–d) measured 27 × 20 × 6 mm, is bright yellow in colour and contains no other inclusions. The wasp was apparently covered in a single flow or drop. Since the appendages of this specimen are not positioned in a certain direction, which would indicate the flow direction, it is clear it was trapped by a resin drop that quickly embedded it completely (compare^1^). The abdomen is bent forward, with the ovipositor pointing toward the ventral part of the thorax. One side of the wasp is covered in bubbles and milky coatings, while the amber on the other side is clear. The bubbles and also the head and thorax of the specimen, contain rod-like structures visible in the micro-CT scan. Some of them are chaotically piled up, while others are parallel and next to each other. The outside of the specimen shows no signs of destruction, however the scan revealed that it is predominantly an empty cast (Fig. 6).

The amber piece of *Xorides*? *romeo* (Fig. 4e–f) measured 26 × 22 × 5 mm, is bright yellow in colour and contains a eusyninclusion of a mite (Supplementary Fig. 1a–b), some plant matter (Supplementary Fig. 1a + c) and various debris, of which some particles could be frass (compare^21^). Once more, the wasp was probably covered in a single flow. The wings are folded dorsally on the back, and legs are bent. The specimen's sternites on the abdomen, which are weakly sclerotized, appear collapsed, which is clear evidence that the specimen was dead and dehydrated before embedding. This specimen is hollow, incomplete, and the apical part of the abdomen missing; the face and a great part of the thorax are indiscernible. The specimen is surrounded by bubbles, foam, and unidentified particles. We can categorize the bubbles on this fossil into three distinct groups, each with a different origin. First, there are various small bubbles distributed along the body. Secondly, we can see that there are larger structures on the face that look like densely packed with bubbles and appear to be firmly attached to the face. Thirdly, there is an accumulation of various-sized transparent bubbles on the thorax, which is covered with a milky coating. This milky coating is distinct from the two phygadeuontine species, as it does not appear directly on the surface of the wasps’ body, but rather surrounds the densely clustered bubbles and debris.

## Discussion

### The wasps’ last moments, taphonomically inferred

Both phygadeuontine wasps are relatively small in size and they might likely have lacked the strength to fight against the surface tension of the viscous resin. In both specimens, we do find evidence that they were alive when being embedded in resin. In *Osparvis aurorae*, there are slight traces of movement around the legs and wing base, indicated as roundish smears.The inclusion of *Osparvis aurorae* exhibits milky coatings around the spiracles, which are frequently reported in Baltic amber^22^. Such coatings indicate chemical reactions of exuding body fluids from decaying organisms with resin in early diagenesis^1^. This implies that the specimen in question was not entirely desiccated when being embedded in resin.

The forward-bent abdomen of *G*. *harveydenti*’s clearly indicates that the specimen has died within the resin^23^. *Grana harveydenti* exhibits a discrepancy between the two lateral sides, with the right side clearly visible through transparent amber, while the left side of the specimen is covered with bubbles and milky coating on almost its entire length. The specimen was probably attached on the left side at first. Subsequently, the body fluids interacted with the resin during the early stage of embedding where the surfaces were touching. It is plausible that the chemical reaction between the resin, the body fluids, along with the presence of microbiota producing gases, led to internal pressure and eventually caused the burst on one side. Additionally, observations of pseudoscorpions in amber, which show the milky coatings ventrally and were likely trapped by walking into the resin^26,27^. These pseudoscorpions were either slowly completely embedded or only later covered with a second flow or resin. However, in the case of *G*. *harveydenti* only one resin flow is visible, and the white coating does not appear to affect the outer appearance of the specimen, but only the visibility through the amber.

Additionally, we observe various bubbles on the left side of *G. harveydenti*. One exits from the tentorial pit on the face, larger ones are found along the thorax and abdomen, and smaller ones on the fore leg and beside the wings. Only the bubbles next to the wings are not in direct contact with the body, and given that bubbles typically ascend, this suggests the wasps’ dorsal side was facing upwards (Fig. 8d).

Since both Phygadeuontinae seem to have died inside the amber, the question remains why one is so well preserved, also internally, and the other one is a hollow cast. A number of factors may influence the degree of preservation observed in fossils. Amber is known to possess antimicrobial properties against bacteria and fungi^28,29^, but just the sealing of an organism in resin does not guarantee its protection from decomposition; it is rather the chemical structure of the sealing medium itself^3^ or what is sealed inside and reacts with the resin, that determines the degree of protection afforded. Baltic amber is probably not originating from a single plant source^30,31^, and the amber could thus differ in chemical structure^25,30^. Chemical properties of resin, which can vary in trees within a population^31^can greatly affect the preservation of insect inclusions^3,32^.

Another factor that may influence the speed of decomposition of inner structures between specimens is the presence of different gut microbiota. It has been demonstrated that small variations, possibly influenced by variable food availability, can significantly impact the preservation of the specimens^3^. Consequently, preservation differences can even occur among specimens fossilized in the same amber piece^33^. It has been hypothesized that when a specimen has not recently consumed food, it might have fewer gut microbes^3^, potentially leading to better preservation. In the case of *Osparvis aurorae* it is possible that the specimen had a relatively empty stomach, which may have contributed to the well-preserved inner structures and organs. *Grana harveydenti* on the other hand, may have consumed food before becoming embedded in resin, which resulted in the mostly hollow cast. The rod-like structures of *Grana harveydenti*, which are only visible in the micro-CT scan, are difficult to assign. It is possible that these structures represent dried muscle tissue that was relocated after the specimen burst open on the side from internal gases. This is because muscular tissues are more likely to be preserved than other proteinaceous organs^34^. However, these structures seem arranged too chaotically for muscle tissue, but are also too large for being microbes.

**Figure 7:**
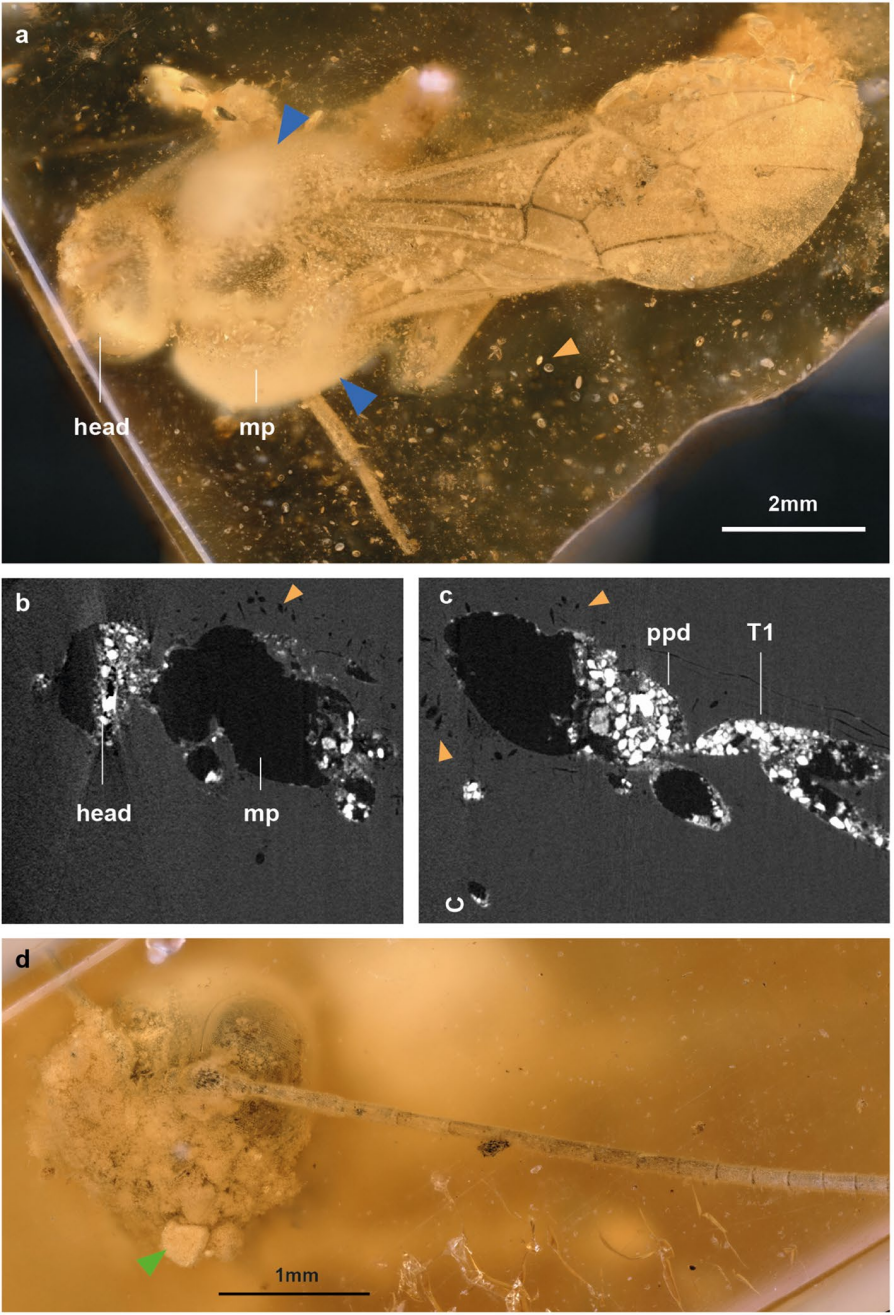
Taphonomy of holotype of *Xorides*? *romeo* sp. nov. (**a**) Photo of complete specimen, in dorsal view, with most of the head and mesopleuron (mp) hidden behind a dense cloud of more transparent bubbles of various sizes with an additional layer of milky coatings over it (blue triangles). Orange triangles indicate the various debris found in the amber piece. (**b**, **c**) Slide of micro-CT scan showing the head, mesopleuron, propodeum (ppd) and first tergite (T1). (**d**) Photo of the head from front view, with clustered structures containing miniscule bubbles (green arrow) on the face.

In *Xorides*? *romeo*, the posture is quite specific, with the wings folded on the back and the legs bent (Fig. 7a), which does not align with the hypothesis that the specimen struggled to escape. If alive, it would have been capable of freeing itself from the sticky tree resin (compare^35^).

The legs are partially missing, indicating that they were disarticulated prior to the specimen being embedded. In conjunction with the partially damaged exoskeleton on the head, thorax, and abdomen (Fig. 7b–d), it is reasonable to posit that the wasp was dead for a while before being covered in resin. It is very difficult to ascertain the underlying causes of the various effects observed in this specimen during the decomposing process. What caused the milky coating around the bubbles on the thorax is difficult to say, but it might be caused by a reaction of resin and the moisture of various debris that is surrounding the specimen. Our findings of a pre-entombed death are corroborated by previous experiments in resin, which demonstrated that specimens that died and dehydrated in isolation from resin exhibit the most pronounced decomposition^3^.

Taxonomy and morphology revealing ecology.

*Grana*, as well as probably *Osparvis*, belong to Phygadeuontinae, which is the most abundant ichneumonid subfamily in Baltic amber and is also very common in temperate forests today^16,20^. Phygadeuontinae are idiobiont ectoparasitoids of a diverse range of hosts that are more or less concealed^18^. They parasitize insect pupae and prepupae, spider egg sacs, and Diptera larvae and puparia, sometimes acting like a pseudohyperparasitoid of Lepidopteran hosts^20^. Based on our current understanding of extant Phygadeuontinae, there is no evidence of direct association with wood-boring larvae^36^. -Nevertheless, numerous genera (e.g. *Aclastus* Förster, 1869, *Gelis* Thunberg, 1827, *Isadelphus* Förster, 1869) can be observed on tree bark, searching for hosts that use bark crevices or moss on trees as a safe place for pupation. Furthermore, there are additional direct links, for instance in *Tropistes* Gravenhorst, 1829, which attacks Raphidioptera under tree bark, or *Obisiphaga stenoptera* (Marshall, 1868), which attacks egg sacs of pseudoscorpions. Both of these hosts are also found in Baltic amber^26,27,37^. Very occasionally, fertilized females can be found under tree bark during wintertime, where some species apparently hibernate as adults (unpublished data;^38^). However, there has been little research on this phenomenon within this subfamily, and it remains unclear whether diapause was an existing mechanism within the warm temperate resinous Forest.

**Figure 8:**
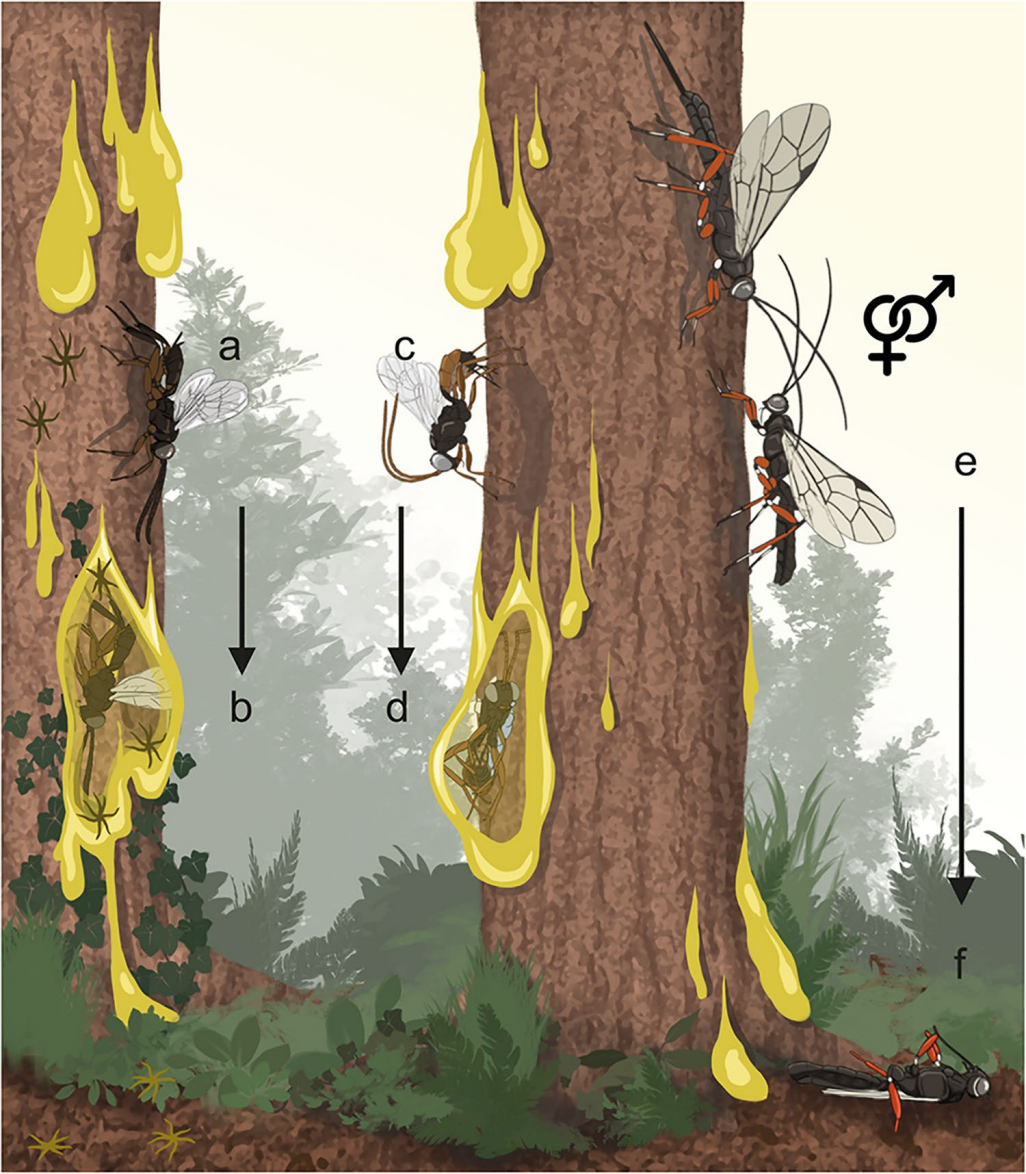
Speculative scenario of the last moments of three Darwin wasps in the resinous forest, which produced Baltic amber. (**a**) *Osparvis aurorae* alive, and (**b**) entrapped in a resin flow, together with oak trichomes. (**c**) *Grana harveydenti* alive, and (**d**) entrapped in a resin flow, with air bubbles. (**e**) *Xorides*? *romeo* looking for a freshly emerged female. (**f**) Dead male *Xorides*? *romeo* After its death, it fell on the forest floor below a resin-producing tree, where it was probably only later fully covered with resin. Colours of the specimens are based on colour patterns of extant species of the respective genus (*Xorides*) and subfamily (Phygadeuontinae). Sizes are not correct but adjusted for visibility on the figure.

The morphology of both phygadeuontine fossils does not provide any evidence for a close association with trees either. However, they could be suitable for a life in the canopy or undergrowth of the resinous forest (i.e. ‘the humid lower shrub-herbaceous layer of the forest’^39^. In other words, both specimens may have been trapped landing by accident on the tree resin or were surprised by extensive resin flow when resting on tree bark (Fig. 8a–d). An important morphological indication for a particular host usage is the ovipositor; which can be identified by length, shape, and structure of the tip. Both species lack a subapical notch of the dorsal valve typically found in endoparasitoid Darwin waps^40^, which supports the hypothesis that they probably were ectoparasitoids. In *Osparvis aurorae*, the ovipositor is slightly downcurved and rather short. This fossil species displays a rather robust habitus and the first tergite is equally robust. Especially in combination with the shorter ovipositor, it is rather unlikely that the metasoma was highly mobile (in contrast^39^), which is consistent with species from the subfamily, which are typically adapted to more or less immobile hosts. The habitus of *Osparvis aurorae* (stout appearance, firmer legs, shorter and downcurved ovipositor) could suggest an association with a more exposed host, such as a lepidopteran or coleopteran pupa in a thin cocoon, or spider egg sacs, hidden below the tree bark, but also solitary aculeate wasps, which nest in dead wood. The ovipositor of *Grana harveydenti* is lanceolate in shape and of average length for Phygadeuontinae. This species is more slender than *O. aurorae*, although the first tergite is also quite robust. *Grana harveydenti*’s rather average phygadeuontine habitus is similar to species of *Xenolytus* Förster, 1869, *Stibeutes* Förster, 1850 and *Orthizema* Förster, 1869, all extant genera that parasitize Lepidoptera, one of the main host groups of Phygadeuontinae. Nevertheless, as *G. harveydenti* lacks specific traits that would narrow the range of potential hosts to make clearer assumptions, it is possible that this species could be linked to any kind of host.

Xoridinae species are primarily associated with wood-boring beetles with particular reference to Cerambydicae, and to a lesser extent Buprestidae, and their larvae develop as idiobiont ectoparasitoids on their hosts^36^. With regard to the hosts and host trees, some species are more specialized than others. Both conifers and deciduous trees are known to be used by extant species^41^. The production of resin can serve as a defense mechanism against insects that may otherwise cause further damage to the tree. This is particularly relevant in the case of wood-boring beetles, which may be deterred by the resin^1^. Reconstructions of the resinous forest, which produced Baltic amber, typically emphasize the presence of conifers^15^, but there are also important indications from preserved pollen or through the analysis of certain species groups (i.e. an overview of the known records of Cerambycidae^42^), which rather suggest a mixed forest, possibly quite similar to a temperate, middle-European forest. In ichneumonids whose hosts are wood-boring beetles, males typically emerge earlier and aggregate on the tree bark around spots where females are about to emerge^36,43–45^ (FV personal observations). In current temperate zones, species can be observed roughly from April to September (for some species suggesting either one prolonged or two generations), but emergence times are generally situated around May or June, just before summer (e.g. ^41,46^, GBIF). To explain the presence of the *Xorides*? *romeo* male, we can hypothesize that it was flying around a tree, waiting for a chance to mate with a female and that it died perched on tree bark or at its foot (Fig. 8e–f). The location on the floor would also help to explain the presence of considerable quantity of debris around the specimen.

A male bias among certain groups of Darwin wasps found in Baltic amber has been previously noted by Manukyan^39^. This suggests that certain male wasps were more actively flying, in search of a mate. Another potential explanation is that some species are attracted to the coloration or scent of resin which is also found in beetles^47^. Male specimens found in Baltic amber from extant subfamilies such as Rhyssinae^17^, Poemeniinae^48^, and Xoridinae, which are associated with wood-boring hosts could be attracted to the resin scent because they link it to emerging females. The hosts of the fossil subfamily Pherombinae, of which there also exists a male bias in Baltic amber, are not known; however, it is possible that they were wood-related holometabolous insects.

## Conclusion

Taphonomic processes offer insights into past environments and ecosystems in which the organisms lived and died. Although the reasons and mechanisms behind the entrapment of certain organisms in the fossil resin remain poorly understood, studies such as ours represent crucial initial steps towards the reconstruction of the paleoecosystem of the resinous forest that produced Baltic amber.

We hypothesize that smaller specimens are better preserved as they are more likely to become trapped alive and get completely covered more rapidly. Through evaluation of the wasps’ body postures, morphology, and taxonomy, we explore the reasons for their presence in amber. Despite the absence of a direct ecological link between the phygadeuontine wasps and the Baltic resiniferous trees, we discussed potential host affiliations based on their general habitus. Conversely, larger specimens such as the *Xorides* male may have been entombed in resin post-mortem. The specimen serves as another example of the male bias observed in larger ichneumonids in amber, which is likely influenced by the fact their hosts are wood-boring insects. The evaluation of taphonomic processes, morphology, and body posture of the wasp species allows us to interpret the stories of these three wasps and serve as puzzle pieces to decipher the ancient ecology and lifestyle of extinct species.

## Material and methods

### Fossil material

To show the varying degrees of taphonomic traces in Baltic amber, we studied three new fossil specimens that are deposited in the collection of the Natural History Museum in Basel (NMB, Switzerland). We have acquired the amber specimens from reputable commercial dealers whose practices are consistent with local and international legal guidelines. The holotypes of *Grana harveydenti* gen. et sp. nov. and *Osparvis aurorae* gen. et sp. nov. were acquired from the Baltic amber shop amberinclusions.eu, run by Jonas Damzen. The holotype of *Xorides*? *romeo* sp. nov. was acquired from the Baltic amber shop ambertreasure4u.com, run by Marius Veta. All three amber pieces were mined in Yantarny mine, in the Kaliningrad region in Russia. The origin and exact age of the amber in the Kaliningrad region are difficult to estimate since it includes different localities with different stratigraphic layers^49^. Nevertheless, the deposits are Eocene in age, most likely Late Eocene^15^.

### Micro-CT scanning

We performed Microtomography scans at the Core Facility "Micro- and Nanotomography'' of the University of Basel (Switzerland) using a phoenix|x-ray nanotom® m (Waygate Technologies Wunstorf, Germany). We then segmented the CT scan data in AVIZO Ⓡ 7.0 software (Visualization Sciences Group). A voxel size of 4 μm was used in *Osparvis aurorae* gen. et sp. nov. and *Grana harveydenti* gen. et sp. nov. while the larger specimen *Xorides*? *romeo* sp. nov. was scanned with a voxel size of 6.5 μm. The 3D models of the micro-CT scans can be found in the repository MorphoBank (http://morphobank.org/permalink/?P5223).

### Photos, illustrations, and descriptions

The amber pieces were immersed in a sugary solution to increase the visibility of the fossil inclusion^25^ and then photographed with a Keyence VHX 600 camera system with a magnification of 100–200.

To create interpretative illustrations of each specimen, we used several detailed images and the 3D models as templates in Adobe Photoshop. Uncertain interpretations of body characteristics are indicated with dotted lines in the drawings.

For the species descriptions, we took the measurements directly from the 3D model, visualized in MeshLab^50^. Metasoma length was measured from the base of the first tergite to the metasoma apex, without measuring the ovipositor.

### Terminology

For the terminology in the descriptions, we followed Broad et al.^20^, except for wing venation, which follows mostly Spasojevic et al.^51^.

The three fossil species were compared with the specific morphological characters of subfamilies and genera from different references^20,52–54^. For the narrower taxonomic comparison within the groups of Phygadeuontinae, we refer to the tribes and subtribes defined by Townes^52^.

### Supplementary Information

Supplementary Video 1.

Supplementary Figure 1.

Supplementary Information 2.

## Data Availability

All data generated or analyzed during this study are included in this published article and its supplementary information file.
