# Supplementary material for: Three taphonomic stories of three new fossil species of Darwin wasps (Hymenoptera, Ichneumonidae)
Source: Sci Rep. 2024 Jul 29;14:17415. doi: 10.1038/s41598-024-67466-z (PMC11286866; doi:10.1038/s41598-024-67466-z)
Supplement: Supplementary file 2 — Supplementary Figure 1. [file 41598_2024_67466_MOESM2_ESM.docx]

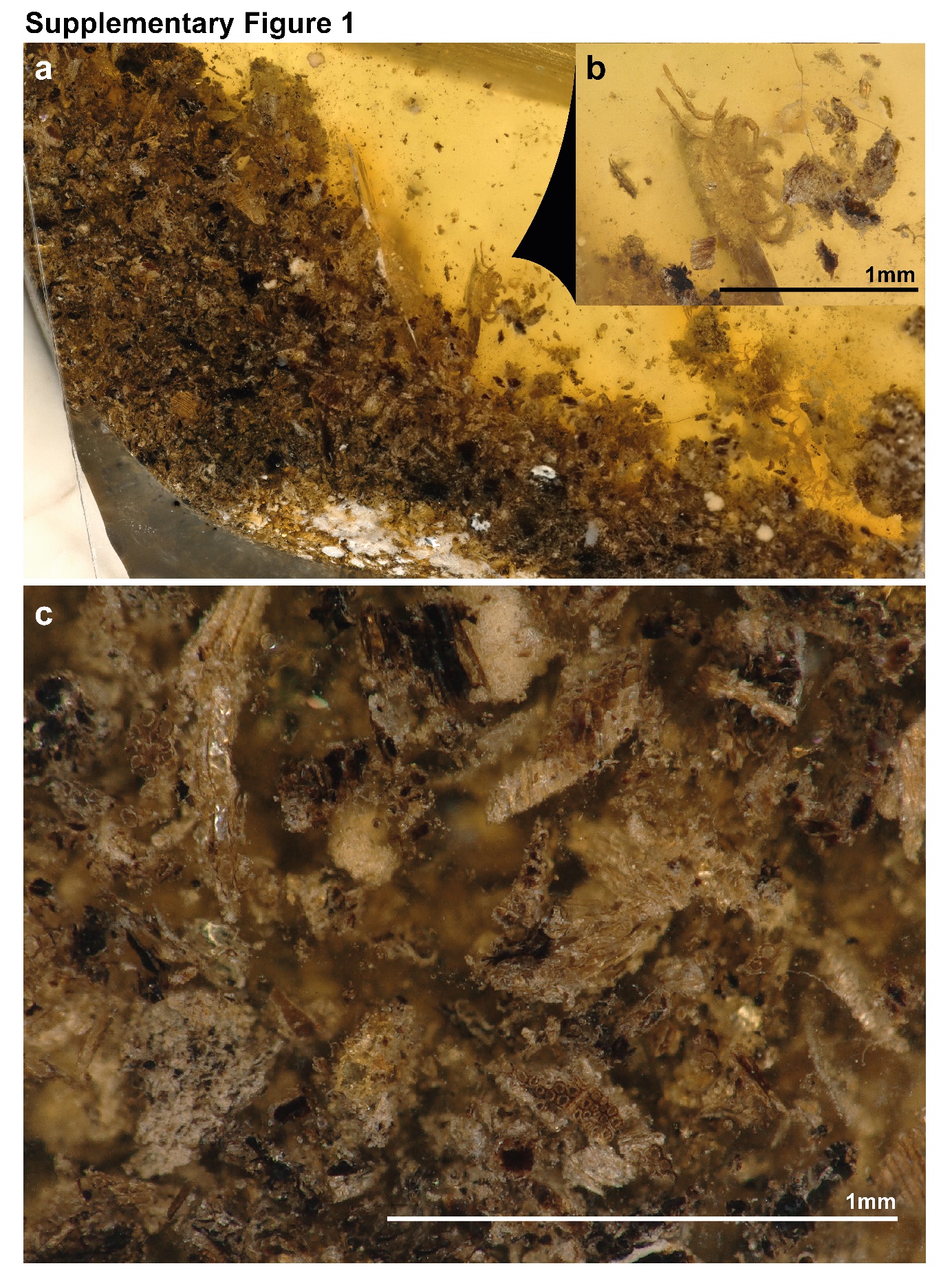


**Supplementary Figure 1**

Eusynclusion of *Xorides*? *romeo* with close-ups. (a) Various debris found in one corner of the amber piece with (b) a mite and (c) plant matter.
