# Supplementary material for: Three taphonomic stories of three new fossil species of Darwin wasps (Hymenoptera, Ichneumonidae)
Source: Sci Rep. 2024 Jul 29;14:17415. doi: 10.1038/s41598-024-67466-z (PMC11286866; doi:10.1038/s41598-024-67466-z)
Supplement: Supplementary file 3 — Supplementary Information 2. [file 41598_2024_67466_MOESM3_ESM.docx]

**Supplementary material – Species descriptions**

Systematic Palaeontology

**Hymenoptera** Linnaeus, 1758

**Ichneumonidae** Latreille, 1802

Subfamily **Phygadeuontinae?** Förster, 1869

Genus ***Osparvis*** Viertler, Schwarz, Verheyde et Klopfstein, gen. nov.

**ZooBank**: urn:lsid:zoobank.org:act:6527F151-3165-40CC-A3C4-D69367981A38

**Type species:** *Osparvis aurorae* gen. et sp. nov.

**Etymology:** Combines the words “os” and “parvum” in Latin, meaning “small mouth” due to the very small mandibles and small clypeus of the specimen. Gender: masculine.

**Systematic placement.** This fossil is difficult to place in a Darwin wasp subfamily, as it combines several plesiomorphic features with characters that are very rare or even unique in Darwin wasps. Among the ancestral characters are the outward-bowed vein 2m-cu with two bullae in the forewing, the completely areolated propodeum, and finally the medium-length ovipositor with nodus and ventral ridges, all characteristics that were already present in wasps from the Cretaceous^1^. Rare characters are the small clypeus, exposed labrum and long malar space, all features only present in very few species (compare ^2^). The rather broadly attaching first tergite with the spiracle slightly posterior to the middle and a large laterotergite, on the other hand, is unique in Darwin wasps.

The fossil shares characteristics of some Phygadeuontinae, such as two bullae on vein 2m-cu, the presence of a sternaulus, absence of a glymma, completely areolated propodeum and the ovipositor shape (compare ^2,3^). However, it also combines some characteristics that are all rather rare in Phygadeuontinae, such as an exposed labrum and an open areolet that looks rather quadratic than pentagonal. The tergite 1 is another characteristic that is very special in this fossil with a very stout appearance and an angle posteriorly with the spiracle only slightly behind the middle. Those characteristics are ambiguous for the subfamily placement, but nothing really speaks against placing it in Phygadeuontinae. Therefore, we are tentatively place it in this subfamily.

**Discussion of alternative placements.** The fossil specimen also shares morphological similarities with Adelognathinae, Orthopelmatinae and Orthocentrinae. With the exposed labrum, the spiracle of the first tergite slightly posterior to the middle and the small size the fossil resembles Adelognathinae. However, the fossils higher number of antennal segments and sternaulus is not found in Adelognathinae. Its clypeus, exposed labrum, and wing venation resemble Orthopelmatinae, but it differs in several key aspects such as the absence of a longitudinal furrow across the mesopleuron, the shape of the first tergite and sternite, and the morphology of the ovipositor.

It also resembles some Orthocentrinae with the clypeus shape and the converging mandibles. However, it can be distinguished from the *Orthocentrus*-group due to the absence of elongated scapes and specific facial features like a strong protrusion, no clypeal sulcus, and the eyes not strongly converging.

**Further comments to diagnosis:** Genera that also possess a sclerotized first laterotergite, like Dichrogaster Doumerc, 1855, Neopimpla Ashmead, 1900, Anurotropus Cushman, 1924, and Gnypetomorpha Förster, 1869, do not have an exposed labrum. The thickened antenna and relatively short mandible, which is strongly tapered towards the apex, can be found in some genera previously placed in Acrolytina. However, the overall proportions of the fossil specimen are rather compact, the head very large and strongly narrowed behind the eye, and the first tergite is short and anteriorly rather wide, which is rare in Acrolytina. The only exception is Neopimpla Ashmead, 1900, which also has an open areolet. However, Neopimpla has large thyridiae, narrow laterotergites, and broad transverse impressions on tergite 2, which is not the case in the fossil.

The fossil differs from the two extinct phygadeuontine genera Madma Viertler, Klopfstein, Jouault & Spasojevic 2022 and Magnocula Viertler, Klopfstein & Spasojevic 2023 by its stout body, open areolet, and enlarged laterotergite 1. Also, both these genera lack a bilobed clypeal apex, exposed labrum, and broad first tergite.

**Description:** Malar space length about mandibular width at base. Labrum clearly exposed below clypeus. Clypeus small, laterally slightly convex. Mandible bidentate, strongly tapered, both teeth of about same length and width. Maxillary palp with five segments, labial palp with four segments. Epomia complete. Mesoscutum evenly shagreened, no modification on base of notaulus, notauli converging and extending to the middle. Epicnemical carina vertical to around mid-height of pronotum. Sternaulus distinct anteriorly. Metapleuron with juxtacoxal and pleural carinae present. Propodeal carination complete. Hind coxa of normal dimensions, slightly longer than wide. Tarsal claws simple. Areolet open. 2m-cu with two bullae. Hind wing with 2CU + cu-a not intercepted. Tergite 1 stout, laterally angled in posterior part, as long as posteriorly wide, spiracle slightly posterior to middle; with distinct dorso-lateral carina and latero-median carina almost reaching posterior end of first tergite; no glymma; large triangular laterotergite. Tergite 7 similar in size to tergite 6. Ovipositor sheath with dense, short pubescence. Ovipositor parallel sided, with nodus and fine ridges or teeth on ventral valve.

*Osparvis aurorae* Viertler, Schwarz, Verheyde et Klopfstein, sp. nov. (Figure 5)

**ZooBank**: urn:lsid:zoobank.org:act:CCB60345-F4D1-4B24-B06C-75CDCA65A9AA

**Etymology:** Named after the fictional character Aurora from the Walt Disney movie “Sleeping Beauty”, since this specimen is very well preserved.

**Type specimen:** Holotype: female (NMB F3847). Baltic amber. Provenance: Kaliningrad region (Yantarny, Russia), Late Eocene. Deposited in Switzerland, Natural History Museum in Basel. syninclusion of some oak flower stellate hairs (trichomes).

Diagnosis. See genus diagnosis.

**Description:** Body 4.4 mm. Color difficult to interpret, but head and mesosoma appear entirely dark.

**Head.** Sculpture smooth, rather large compared with mesosoma and metasoma, in frontal view wide and short. Mandible moderately large, base width 2× apex width, bidentate with both teeth similar in length and width, not twisted. Malar space around 1.1× mandibular width at base, with distinct impression. Labrum clearly exposed, only slightly smaller than clypeus. Clypeus small, convex laterally, apex weakly concave or bilobed in front view and 2× as wide as high. Face separated from clypeus by what seems a weak clypeal sulcus, flat with weak median swelling; tentorial pit strongly enlarged. Eye moderately large, 0.76× as high as head in lateral view; inner margins of eyes parallel. No modification between antennal sockets and on frons. Ocelli of normal size. Vertex short, distance between lateral ocellus and occipital carina less than diameter of ocellus. Occipital carina complete. Flagellum 3.2 mm, with 20 segments; first segment 2.5× longer than wide; after 5^th^ segment wider than long; tip of apical flagellomere appears unmodified.

**Mesosoma.** Pronotum 0.66× as long as high; anterior margin simple; epomia complete. Mesoscutum appears shagreened; notaulus distinct to about half the length of the mesoscutum, notauli converging but not reaching each other. Scutellum convex. Metanotum flat. Mesopleuron sculpture smooth, slightly convex, with epicnemial carina vertical, reaching to around mid-height of pronotum. Mesosternum behind fore coxa simple. Posterior transverse carina of mesosternum either complete or present but absent in front of middle coxa. Sternaulus distinct anteriorly, then rather weak from middle to posterior end. Metapleuron about as wide as high; with submetapleural carina complete, no modification in anterior section; pleural carina complete, juxtacoxal carina present but partially reduced. Propodeum shagreened with some weak rugae, short, rounded, with complete carination, spiracle on anterior transverse carina and closer to lateral longitudinal carina than to pleural carina. Dorsal margin of metacoxal cavities above ventral margin of metasomal cavity. Legs simple. Front femur including trochanter and trochantellus 0.34× length of fore wing. Mid tibia with two spurs, equally long. Hind coxa of normal dimensions, slightly longer than wide; hind femur 3.1× longer than wide, with widest part basally; tibia with two spurs of equal length, with a fringe of parallel setae on inner side that looks similar to other pubescence; first tarsomere 5.3x longer than apically wide. Tarsal claws simple and rather short.

**Wings.** Fore wing 2.8 mm. Areolet open, with 2+3M 1.8× 2Rs. 2m-cu bowed outwards in slightly sinuous curve; with two small bullae, covering around 25% of 2m-cu. 4Cu 1.2× 5Cu. 4Rs slightly bowed. 1cu-a postfurcal to M1 + 1Rs, emerging in right angle. 5M tubular throughout. 1m-cu & 2Rs+M slightly bowed, with ramulus absent. 3Cu about same length as 2cu-a. Pterostigma 3.8× longer than wide, same length as 1R1. Cell 2R1 2.5x longer than wide. 2Cu 0.6× 1M + 1Rs, 0.8× r-rs. 1M + 1Rs 1.4× r-rs. Hind wing with M+Cu slightly bowed on entire length. 2CU + cu-a (nervellus) not intercepted. 1Rs 0.75× rs-m, 2Rs partially spectral.

**Metasoma.** Depressed. Tergite 1 stout, angled in posterior 0.3, around as long as wide, slightly tapering in dorsal view; bowed or slightly angled at spiracle position in lateral view; with dorso-lateral longitudinal carina reaching beyond middle of tergite 1, above spiracle; spiracle slightly posterior to middle; latero-median carina distinct on entire length, more or less parallel; no glymma; laterotergite large, triangular, strongly sclerotized and starting just before spiracle of tergite 1. Sternite 1 0.4× tergite 1. Tergite 2 subquadrate, with no latero-median carina; laterotergite partially folded under, appears narrow. Laterotergite of tergite 3 partially folded under, appears narrow. Tergite 4**–**7 less than half the length of tergite 1**–**3. Laterotergite of tergite 4 moderately broad, 2.5× longer than wide. Tergite 7 similar in size to tergite 6. Tergite 8 short. Sternite 6 transverse. Ovipositor sheath with dense, short pubescence; parallel sided; 0.24× metasoma length. Ovipositor subcylindrical, parallel sided, dorsal valve with nodus, and with fine teeth or ridges.

Subfamily **Phygadeuontinae** Förster, 1869

Genus ***Grana*** Viertler, Schwarz, Verheyde et Klopfstein, gen. nov.

**ZooBank**: urn:lsid:zoobank.org:act:A1D71E27-639C-4C4F-AFB1-64207D0E3244

**Type species:** *Grana harveydenti* sp. nov.

**Etymology:** Short form of the Latin adjective “granulosus”, which emphasizes the overall granulate body sculpture of the specimen. Gender: feminine.

**Systematic placement.** The combination of the fossil’s body characteristics clearly points to Phygadeuontinae, with the presence of a sternaulus that ends above the posterior ventral edge of the mesopleuron, the fore wing with a pentagonal areolet and two bullae on vein 2m-cu, the complete carination of the propodeum, the petiolate first tergite with the spiracle behind the middle, and the rather long ovipositor^2^.

**Discussion of tribal placement.** The fossil resembles *Gelis, Formocryptus*, and *Xenolytus* within the subtribe Gelina, with a subbasal swelling of the mandible, a similar apical truncation of the scape, two bullae on 2m-cu, an intercepted nervellus, the second laterotergite separated from its dorsal part by a crease, and a stout ovipositor. *Gelis* can be excluded because of the fossil’s strong epomia, *Formocryptus* differs by having teeth on the apical margin of the clypeus, and *Xenolytus* can be excluded since the fossil’s gena is much narrower, has the nervellus strongly inclivous and the body extensively granulated.

The fossil also shares general characteristics with genera in Phygadeuontina, with a rather large head, a weakly convex clypeus, similar apical scape truncation, propodeum with complete carination, and an intercepted nervellus in the hind wing. However, within this subtribe many extant genera can be excluded because the fossil’s clypeus has no teeth or tubercles and the area superomedia is rather wide. The fossil has the occipital carina joining the oral carina above the mandible base, weak propodeal apophyses and a lanceolate ovipositor tip, all characteristics that agree with *Stibeutes*. However, generally, *Stibeutes* does not have such an extensively granulated body sculpture and has its clypeus clearly broader than the fossil specimen. There is an atypical extant species in this genus, *Stibeutes blandi* Schwarz & Shaw, 2011, which has a narrower clypeus and a similar granulated body sculpture as the fossil specimen. However, *S. blandi* does not have such a wide head shape and has several metasomal segments with a striate sculpture.

**Further comments to diagnosis:** When comparing the fossil specimen with extinct groups, only one Phygadeuontinae taxon has been described so far from Baltic amber^4^, despite this being the most commonly found subfamily^5^. The fossil differs from the extinct genus Magnocula, which is defined by a carina on the base of notaulus, a petiolate quadratic areolet, a slender first tergite without a distinct latero-median carina and the first sternite reaching almost to the posterior end of the first tergite, and a slightly elongate eighth tergite, all characters not present in our fossil.

The other extinct phydadeuontine genus Madma was found in Lowermost Eocene Oise amber^6^ and shares the bilobed posterior transverse carina on the mesosternum with the new fossil specimen. However, our fossil lacks an apical tooth on the fore tibia, has no elongated pentagonal areolet, and the latero-median carina does not reach the posterior end of the first tergite. This fossil specimen exhibits characteristics from different phygadeuontine genera that are not found in this combination, therefore we describe a new genus.

**Description:** Granulate sculpture on head, mesosoma and metasoma. Malar space length shorter than mandibular width at base. Labrum concealed. Clypeus separated from face, apical margin convex and without teeth or tubercles. Occipital carina complete. Maxillary palp with five segments, labial palp with four segments. Epomia strong and complete. Posterior transverse carina of mesosternum incomplete, with two extending broader lobes. Sternaulus distinct. Juxtacoxal carina and pleural carina present. Propodeum with complete carination. Hind coxa of normal dimensions, clearly longer than wide. Fore wing with closed, pentagonal areolet. 2m-cu with two bullae. Hind wing with nervellus intercepted below the middle. Tergite 1 petiolate, elongate, with latero-median carina and no glymma. Laterotergite on tergite 1 triangular. Laterotergites of tergite 2 and 3 broad. Ovipositor sheath with short, dense pubescence; parallel sided. Ovipositor tip lanceolate, with weak teeth or transverse ridges on ventral side.

*Grana harveydenti* Viertler, Schwarz, Verheyde et Klopfstein, sp. nov. (Figure 6)

**ZooBank**: urn:lsid:zoobank.org:act:D9592537-3ED5-4D69-A981-C0C074BB2CE0

**Etymology:**  Named after the fictional supervillain Harvey Dent (also called Two-face), from the DC-Comic Batman. Like this character, this specimen has one side of its body destroyed, while the other side is intact.

**Type specimen**. Holotype: female (NMB F3848). Baltic amber. Provenance: Kaliningrad region (Yantarny, Russia), Late Eocene. Deposited in Switzerland, Natural History Museum in Basel.

**Description:** Body, 4.5 mm. Color difficult to interpret but appears dark.

**Head.** Rather short, granulated sculpture. Mandible moderately large, bidentate, base 2× width of apex, teeth similar in length and width, or lower slightly shorter, not twisted. Malar space between 0.8**–**0.9× mandibular width, smooth, without subocular sulcus. Clypeus in frontal view convex, 2× as wide as long, subquadrate in shape, separated from face by weak clypeal sulcus, apical margin without teeth or tubercles. Apical tentorial pit normal sized. Face slightly convex with weak median swelling. Eye large, in lateral view 0.87× head height, inner orbits parallel throughout. No modification visible between antennal sockets. Ocelli of normal size. Vertex weakly and evenly rounded down to occipital carina. Scape with 40 degrees apical truncation from transverse, 1.6× longer than wide (measured from front view: longest length, widest width). Pedicel smaller than scape. Flagellum 3.3 mm with 21 segments, first segment 4× longer than wide at base, then getting shorter and apical ones almost as long as wide.

**Mesosoma.** Sculpture granulated. Pronotum 0.65× as long as deep; anterior margin simple; epomia strong and complete. Mesoscutum extensively granulated; notauli slightly converging, reaching middle of mesoscutum. Scutellum convex, without lateral carina. Metanotum short, appears triangular in lateral view. Mesopleuron flat, with complete epicnemial carina almost reaching subtegular ridge. Mesosternum behind fore coxa simple. Posterior transverse carina of mesosternum incomplete, present in middle with two extending broader lobes reaching to about half width of mid coxa, then absent laterally. Sternaulus distinct, ending above mid coxa posteriorly. Metapleuron about as wide as high; with submetapleural carina complete, no modification on anterior section; with juxtacoxal carina and pleural carina present and complete. Propodeum short, lateral length 0.7–0.8x height and rounded, with complete carination, spiracle on anterior transverse carina and closer to lateral longitudinal carina than to pleural carina; well defined area superomedia, about as wide as long. Dorsal margin of metacoxal cavities above ventral margin of metasomal cavity. Legs simple. Front femur, including trochanter 0.29× length of fore wing. Hind leg with coxa 1.6× longer than wide; femur 3.6× longer than wide; tibia with two spurs of equal length; first tarsomere 6.9× longer than apically wide.

**Wings.** Fore wing 3.15 mm. Areolet closed, pentagonal with 2+3M 1.75× of 4M and 3Rs 0.7× of 4M. 2m-cu evenly bent outwards, with two bullae, together covering 25% of 2m-cu. 4Cu 1.9× 5Cu. 4Rs slightly bowed. 1cu-a only slightly postfurcal to 1M + 1Rs, emerging oblique. 5M tubular. 1m-cu & 2Rs+M slightly bowed or angled; ramulus absent. 3Cu about same length as 2cu-a. Pterostigma 2.6× as long as wide, 0.98× 1R1. Cell 2R1 2.2× longer than wide. 2Cu 0.64× 1M + 1Rs, 1.1× r-rs. 1M + 1Rs 1.7× r-rs. Hind wing with nervellus intercepted below the middle and about vertical, 2RS and 3CU tubular throughout, 1Rs 1.15× rs-m; with one basal hamulus.

**Metasoma.** Cylindrical, sculpture granulated. Tergite 1 petiolate, elongate with 1.6× as long as posteriorly wide, gradually widening posteriorly; flat to slightly bowed on whole length in lateral view; spiracle posterior to the middle; strong dorso-lateral and ventro-lateral carinae on whole length; weak latero-median carina dorsally; no glymma; laterotergite triangular, starting on height of spiracle. Sternite 1 flat, 0.52× tergite 1. Tergite 2 subquadrate, without latero-median carina, laterotergite about 3.33× longer than wide, unsure if creased. Laterotergite of tergite 3 2.22× longer than wide. Laterotergite of tergite 4 2.7× longer than wide. Tergite 8 short. Sternite 6 transverse. Ovipositor sheath with short, dense pubescence; parallel sided; 0.33× metasomal length. Ovipositor, parallel sided; slightly compressed; ventral base simple; tip elongate, appears smooth, broader than rest forming lanceolate shape.

**Subfamily** Xoridinae Shuckard, 1840

**Genus** *Xorides*? Latreille, 1809

***Xorides*? *romeo*** Viertler et Klopfstein, sp. nov. (Figure 7)

**ZooBank**: urn:lsid:zoobank.org:act:1243A9AC-CAC2-4823-A142-175177EB0C37

**Etymology:** Named after the male protagonist Romeo Montague from Shakespeare's Romeo & Juliette.

**Type specimen**: Holotype: male? (NMB F3849). Baltic amber. Provenance: Kaliningrad region (Yantarny, Russia), Late Eocene. Deposited in Switzerland, Natural History Museum Basel.

**Systematic placement.** The elongate body shape, together with the wide gena, and relatively small eye point to a wood-boring subfamily. The antefurcal 1cu-a on the fore wing is not common but occurs in some subfamilies of the informal group pimpliformes (i.e., Poemeniinae, Acaenitinae, Diacritinae, Rhyssinae, Pimplinae) and in the subfamily Xoridinae. However, many of those subfamilies can be excluded by specific characteristics that are different or absent in the fossil (e.g. transverse rugae on mesoscutum in Rhyssinae), or their rather stout appearance (Pimplinae). The fossil has simple claws, rather extensive propodeal carination, an open areolet, a straight 2m-cu, and its nervellus intercepted below the middle, which excludes Poemeniinae, Acaenitinae, and Diacritinae. The elongate body, slightly swollen femor, very short 2Rs vein, and rather elongate first tergite point to Xoridinae.

**Discussion of Xoridinae genera.** Xoridinae comprises 4 extant genera. *Odontocolon* can be excluded since the fossil does not possess a ventral tooth on the hind femur. *Ischnoceros* has a median horn on the frons, which is not the case in this fossil. To confidently exclude *Aplomerus*, we would need to see if tergite 1 is fused to sternite 1, a characteristic that is indiscernible in the fossil’s micro-CT scan and in optical view. However, the head and body of *Aplomerus* seem much flatter and the pronotum lacks the epomia, which appears present in the fossil. Many characteristics are in agreement with *Xorides*. However, some important characters are not visible due to the preservation of the fossil, which would confirm the placement. We can neither see chisel-shaped mandible nor oblique grooves on the laterotergite of tergite 2, as would be the case in *Xorides*. Therefore, we place this fossil species in the genus *Xorides* with uncertainty.

**Further comments to diagnosis:** There are currently two other fossil Xorides described. Xorides sejugatus (Brues, 1910) shares many similarities with our new fossil species. However, X. romeo has 1cu-a clearly antefurcal to 1M&1Rs, while it is postfurcal in X. sejugatus. Also, the hind femur and hind tibia of our species are more slender, with the femur 3.9×, and the hind tibia 8.2× as long as wide (in X. sejugatus hind femur 3×, hind tibia 5.8 as long as wide).

It is difficult to compare our specimen to Xorides lambei (Handlirsch, 1911) since the latter is not well preserved and probably distorted. The only thing we can compare are the fore wings, which show some differences: in X. lambei 3Cu and 2cu-a are similar in length, while in our species, 2cu-a is distinctly shorter. In X. romeo, we find 4Rs to be sinusoidal, while the vein in X. lambei seems evenly bowed. Generally, there is not much to compare, since even the visible veins, as 1m-cu & 2Rs+M, differ in shape from right to left forewing in X. lambei in the drawings.

**Description:** Body min. 11.4 mm. Color difficult to interpret. Sculpture not discernible.

**Head.** Antenna very slender, with at least 18 segments, this part 5.5 mm, evenly thick throughout, scape about 1.5× longer than wide, scape truncation almost horizontal, pedicel smaller than scape, first segment 5.3× as long as apically wide, other segments around 2× as long as wide; maxillary palp long, reaching past fore coxa, with five segments; malar space between 0.8**–**1.3× mandibular base width; inner orbits without dorsal invagination, more or less parallel in front view; eye laterally 0.75× head height; no setae on eye. Ocelli normal sized, lateral ocellus about 1.7**–**2.1× its own diameter distance from eye margin. Vertex long, horizontal, abruptly declivous behind occipital carina.

**Mesosoma.** Pronotum laterally as long as deep. Epomia difficult to interpret, but upper part appears present. Mesoscutum coarsely punctate, with punctures separated by their own diameter, evenly pubescent; median lobe laterally protruding lateral lobes; notaulus deeply impressed, extending posteriorly past center, notauli strongly converging. Scutellum more or less flat. Mesopleuron centrally rather flat. Epicnemial carina present, form and length uncertain. Posterior transverse carina on mesosternum appears short and present only in the middle, between the two mid coxae. Sternaulus absent. Metapleuron 1.8**–**1.9× as long as high; anterior section of submetapleural carina unmodified; pleural carina complete. Propodeum about as long as high, without posterior protrusions; both lateromedian and lateral longitudinal carinae present; anterior transverse carina absent, posterior transverse carina complete, angled. Legs simple. Mid tibia with two equally long spurs. Hind tibia with two equally long spurs; hind coxa 1.7× longer than wide; hind femur 3.9× longer than wide; hind tibia 8.2× as long as apically wide, with thickened setae (spike-like); 1^st^ tarsomere 7.3× longer than apically wide. Tarsal claws appear simple.

**Wings.** Fore wing 8.4 mm. Areolet open, 2+3M 2.2× 2Rs. 2m-cu straight with two bullae. 4Cu 1.7× 5Cu. 4Rs slightly sinusoidal. 1cu-a antefurcal to 1M + 1Rs, 1cu-a emerging in right angle. Pterostigma 3.7× longer than wide, 0.9× 1R1. Cell 2R1 4.3× longer than wide. 5M tubular throughout. 2Cu 0.6× 1M + 1Rs, same length as r-rs. 1m-cu & 2Rs + M straight to slightly bowed. 3Cu 2.5× 2cu-a. Hind wing with nervellus intercepted at 0.65, eight apical hamuli.

**Metasoma.** Depressed; tergite 1 dorsally evenly tapering to front, 1.5× longer than posteriorly wide; laterally rounded at base, no glymma discernible, if present then shallow; lateromedian carina reaching middle or beyond, carina converging but still as far apart as distance to lateral margin, spiracle at 0.4 of tergite 1. Sclerotized part of sternite 1 reaching to about half length of tergite 1. Tergite 2 transverse dorsally, laterally tergite 2 length 0.6× tergite 1; laterotergite creased, about 5× longer than wide. Laterotergite 3 and 4 creased, about 3.33× longer than wide.

## References

1. Kopylov, D. S. A new subfamily of ichneumonids from the Lower Cretaceous of Transbaikalia and Mongolia (Insecta: Hymenoptera: Ichneumonidae). *Paleontological Journal* **43**, 83–93 (2009).

2. Broad, G. R., Shaw, M. R. & Fitton, M. G. *Ichneumonid Wasps (Hymenoptera: Ichneumonidae): Their Classification and Biology*. *Handbooks for the Identification of British Insects* vol. 7 (2018).

3. Townes, H. The genera of Ichneumonidae, Part 2. *Memoirs of the American Entomological Institute* 1–537 (1970).

4. Viertler, A., Urfer, K., Schulz, G., Klopfstein, S. & Spasojevic, T. Impact of increasing morphological information by micro-CT scanning on the phylogenetic placement of Darwin wasps (Hymenoptera, Ichneumonidae) in amber. *Swiss J Palaeontol* **142**, 30 (2023).

5. Manukyan, A. R. & Zhindarev, L. A. Fossil Darwin wasps (Hymenoptera: Ichneumonidae) from Baltic amber. *Palaeoentomology* **004**, 637–647 (2021).

6. Viertler, A., Klopfstein, S., Jouault, C. & Spasojevic, T. Darwin wasps ( Hymenoptera , Ichneumonidae ) in Lower Eocene amber from the Paris basin. *Journal of Hymenoptera Research* **45**, 19–45 (2022).
